# Molecular detection ofserotype groups of *Listeria monocytogenes* isolated from gallbladder of cattle and sheep in Iraq

**DOI:** 10.14202/vetworld.2018.431-436

**Published:** 2018-04-07

**Authors:** Hamza Jawad Al-Ali, Mohsen Abd Al-Rodhan, Samer Abdulsahib Al-Hilali, Alaa Hani Al-Charrakh, Ali Muhsin Al-Mohana, Zainab Jaber Hadi

**Affiliations:** 1 Najaf Veterinary Hospital, Najaf Province, Iraq; 2 Department of Clinical and Laboratory Science, College of Pharmacy, University of Al-Qadisiyah, Diwaniyah, Iraq; 3 Department of Microbiology, College of Medicine, University of Kufa, Kufa, Iraq; 4 Department of Microbiology, College of Medicine, Babylon University, Hillah, Babylon Governorate, Iraq

**Keywords:** cattle, gallbladder, *Listeria monocytogenes*, molecular detection, sheep

## Abstract

**Aim:**

This study was designed to investigate the occurrence of serotypes of *Listeria monocytogenes*, an important food-borne pathogen, in gallbladder samples from cattle and sheep.

**Materials and Methods:**

Three hundred samples were collected and screened for the presence of *L. monocytogenes*. The identification of the isolates was confirmed by API-*Listeria* system and by the presence of hemolysin (*hyl*) gene. The isolates were subjected to polymerase chain reaction-based serotype identification with d1 (division 1), d2 (division 2), *glt, mama* (mismatch amplification mutation assay), and *flaA* (flagellin protein) genes.

**Results:**

A total of 8 (2.7%) *L. monocytogenes* were recovered from 6 (4.0%) samples of sheep and 2 (1.3%) samples of cattle. All isolates showed positive results with Hly primers. Four isolates carried *d1* gene, did not possess *glt* gene and harbored *mama* gene. The serotypes of these isolates were identified as 4a or 4c. The other 4 isolates carried *d2* gene, 3 of them were positive with the FlaA primers, and hence, determined to be a 1/2a or 3a serotype, and 1 isolate was determined to be 1/2c or 3c serotype.

**Conclusion:**

This study concluded that the presence of 1/2a serotype in gallbladder samples indicates public health risk through cross-contamination of meat at slaughterhouses.

## Introduction

*Listeria monocytogenes* is a Gram-positive, facultative, intracellular bacterial pathogen that causes morbidity and mortality in human and livestock [1]. It is a significant foodborne pathogen due to its widespread distribution in nature [2]. Gahan and Hill [3] have already described the isolation of *L. monocytogenes* from the gallbladder in humans. The species has the important feature of virulence as it can colonize in the gallbladder together with extracellular multiplication, which revealed to the presence of an *L. monocytogenes*-specific gene, termed *bsh*, encoding a bile salt hydrolase [4].

*L. monocytogenes* strains are serotyped according to variation in the somatic (O) and flagellar (H) antigens [5]. Although more than 13 serotypes of *L. monocytogenes* have been described [6], only three serotypes (1/2a, 1/2b, and 4b) source the vast majority of clinical cases [7]. Numerous molecular subtyping techniques have identified two major phylogenetic divisions within the species. Division I consists of serotypes 1/2b, 3b, 4b, 4d, and 4e, and Division II consists of serotypes 1/2a, 1/2c, 3a, and 3c, and a Division III consisting of less common serotypes 4a and 4c have also been identified [8]. Polymerase chain reaction (PCR) has been used for detection of serotype groups of *L. monocytogenes* isolates using specific primers related to Division I/III and Division II genes [5,9].

No information about molecular serotyping of *L. monocytogenes* derived from animal sources is available in Iraq. Therefore, an increased demand to investigate the dissemination of *L. monocytogenes* serotypes exists, and hence, this study was designed to determine distribution as well as serotype of *L. monocytegenes* in gallbladders of cattle and sheep.

## Materials and Methods

### Ethical approval

The approval from the Institutional Animal Ethics Committee to carry out this study was not required as no invasive technique was used.

### Sample collection

A total of 300 bile salt samples were collected from gallbladder of cattle (n=150) and sheep (n=150) from the main slaughterhouse in Najaf province, central Iraq. The specimen collection period was from November 2015 to April 2016. All bile samples were stored at −20°C until analysis. Standard bacterial strain *L. monocytogenes* 10403 s was obtained from central health laboratory, Baghdad, Iraq.

### Isolation and identification of bacterial isolates

The International Dairy Federation method was used for isolation and identification of *Listeria* spp. [10]. All samples were incubated at 4°C for 1 week and centrifuged (Eppendorf 5417R refrigerate centrifuge, Germany) at 6000 rpm for 20 min. A 1 ml of bile salt pellets added into 9 ml of *Listeria* enrichment broth (HiMedia, India) and incubated at 30°C for 48 h. After that, 0.1 ml of the enrichment broth culture was spread on HiCrome *Listeria* agar modified (HiMedia, India) and incubated at 37°C for 24-48 h. The isolates were identified to the level of species according to standard microbiological methods [11] and confirmed by API *Listeria* test (BioMerieux, France). The colonial morphology and biochemical tests of *Listeria* isolates were compared with standard *L. monocytogenes* strain 10403 s.

### Molecular detection of serotype groups of isolates by PCR

*L. monocytogenes* DNA extraction was done using Genomic DNA Mini Kit (Geneaid, USA). DNA templates were subjected to PCR using one set of primers targeting *hly* gene and six sets of primers targeting serotypes genes listed in Table-1.

**Table-1:** The primers with their sequences and product size (5).

Primer name		DNA sequence (5×3×)	Product size (bp)
D1^a^	F	CGATATTTTATCTACTTTGTCA	214
	R	TTGCTCCAAAGCAGGGCAT	
Glt^b^	F	AAAGTGAGTTCTTACGAGATTT	483
	R	AATTAGGAAATCGACCTTCT	
Mama^c^	F	CAGTTGCAAGCGCTTGGAGT	286
	R	GTAAGTCTCCGAGGTTGCAA	
D2^a^	F	GCGGAGAAAGCTATCGCA	140
	R	TTGTTCAAACATAGGGCTA	
FlaA^b^	F	TTACTAGATCAAACTGCTCC	538
	R	AAGAAAAGCCCCTCGTCC	
Hly^c^	F	CGGAGGTTCCGCAAAAGATG	234
	R	CCTCCAGAGTGATCGATGTT	

F=Forward primer, R=Reverse primer,

a 2% agarose gel,

b 1.2% agarose gel,

c 1.5% agarose gel

The reaction mixture contained AccuPower™ PCR PreMix (Bioneer, Korea), which premixed ready-to-use solution containing *Tag* DNA polymerase, dNTP, MgCl_2_, and according to Bioneer procedure (Bioneer Corporation, Korea). The mixture was prepared in 0.2 ml Eppendorf tube with 20 µl reaction volumes. The PCR was performed with PCR system (GeneAmp PCR system 9700, Applied Biosystem, Singapore) at 95°C for 3 min except with *mama* gene, 94°C for 5 min for 1 cycle. For the *hly* gene, 94°C, 1 min; 60°C, 1 min; 72°C, 1 min for a total of 30 cycles, then 72°C for 10 min. For *d1* and *d2* genes, 95°C, 1 min; 60°C, 30 s; 72°C, 1 min for a total of 25 cycles, then 72°C for 10 min. For *glt* gene, 95°C, 30 s; 51°C, 30 s; 72°C, 1 min for a total of 25 cycles, then 72°C for 10 min. For *flaA* gene, 95°C, 30 s; 54°C, 30 s; 72°C, 1 min for a total of 30 cycles, then 72°C for 10 min. For *mama* gene, 94°C, 30 s; 48°C, 30 s; 72°C, 1 min for a total of 32 cycles, then 72°C for 10 min. PCR products were separated by electrophoresis in agarose gel containing ethidium bromide (0.5 mg/mL) and visualized in-gel documentation system (BioDocAnalyze Live, Biometra-biomedizinische Analytic GmbH, Germany).

## Results

### Isolation and identification of bacterial isolates

**Figure-1:**
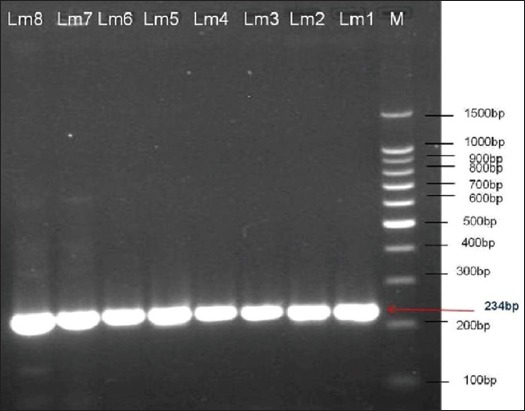
Ethidium bromide-stained agarose gel showing polymerase chain reaction products of *Listeria monocytogenes* DNA amplified with Hlyprimers.The electrophoresis performed at 80 volts for 1.25 h. Lane M, molecular-sized marker (100 bp DNA ladder). Lanes Lm1-Lm8 showed amplification of *hly* gene at 234 bp.

Only 8 (2.7%) of the 300 bile salt samples were found positive for *Listeria* species. The bacteria were present in 4.0% (6/150) of sheep samples and 1.3% (2/150) of cattle samples (Table-2). However, the identification of the suspect colonies was confirmed as they appeared as typical *Listeria* species when they subcultured onto HiChrom *Listeria* agar plates and incubated at 37°C for 48 h. All eight isolates were grown on this medium (typical *L. monocytogenes* colonies) with a special characteristic of blue colonies with a yellow halo (rhamnose fermenting colonies) ranging from 1.5 to 5 mm in diameter. All isolates were confirmed as *L. monocytogenes* using API-*Listeria* tests. In addition, the confirmation process of the *L. monocytogenes* isolates was also conducted by PCR assay to detect the presence of specific virulence trait, *hyl* gene. The isolates were positive with Hly primers (Figure-1). All isolates were obtained separately from November to February.

**Table-2:** Distribution of *L. monocytogenes* in sheep and cattle.

Type of animal	Number of samples	Number of positive samples	Percentage
Sheep	150	6	4.0
Cattle	150	2	1.3
Total	300	8	2.7

### PCR-based serotype identification

**Figure-2:**
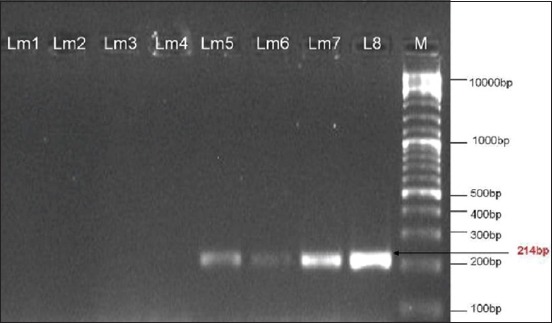
Ethidium bromide-stained agarose gel showing polymerase chain reaction products of *Listeria monocytogenes* DNA amplified with D1primers.The electrophoresis performed at 80 volts for 1.25 h. Lane M, molecular-sized marker (100 bp DNA ladder). Lanes Lm4-Lm8 showed amplification of *d1*gene at 214 bp.

**Figure-3:**
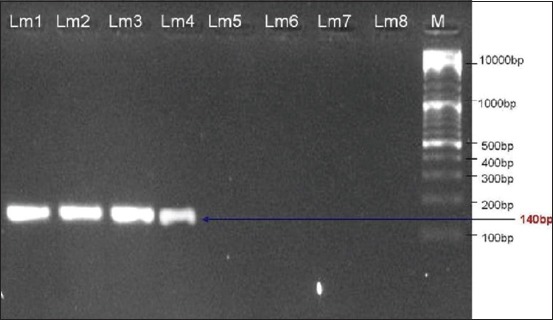
Ethidium bromide-stained agarose gel showing polymerase chain reaction products of *Listeria monocytogenes* DNA amplified with D2primers.The electrophoresis performed at 80 volts for 1.25 h. Lane M, molecular-sized marker (100-10000 bp DNA ladder). Lanes Lm1-Lm4 showed amplification of *d2* gene at 140 bp.

The isolates were tested with D1 primers than with D2 primers. Only 4 (50.0%) isolates (Lm5, Lm6, Lm7, and Lm8) yielded amplification products with the D1 primers (Figure-2), and the other 4 (50.0%) isolates (Lm1, Lm2, Lm3, and Lm4) were amplified with D2 primers (Figure-3).

The four D1 positive isolates were further tested using the Glt primer set. Results revealed that all the D1 positive isolates were negative with the Glt primers. However, negative Glt isolates were tested with Mama-PCR primers to differentiate the serotypes.

**Figure-4:**
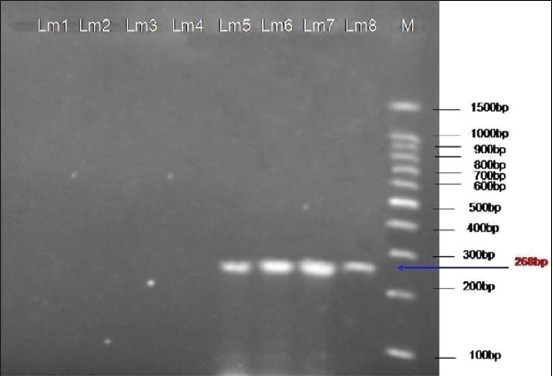
Ethidium bromide-stained agarose gel showing polymerase chain reaction products of *Listeria monocytogenes* DNA amplified with Mamaprimers.The electrophoresis performed at 80 volts for 1.25 h. Lane M, molecular-sized marker (100-1500 bp DNA ladder). Lanes Lm5-Lm8 showed amplification of *mama* gene at 268 bp.

All four isolates gave positive results with Mama primers (Figure-4); therefore, the positive results determined that the serotypes for those isolates were the 4a or 4c serotypes. While all D2 positive isolates were further tested using FlaA primer set.

**Figure-5:**
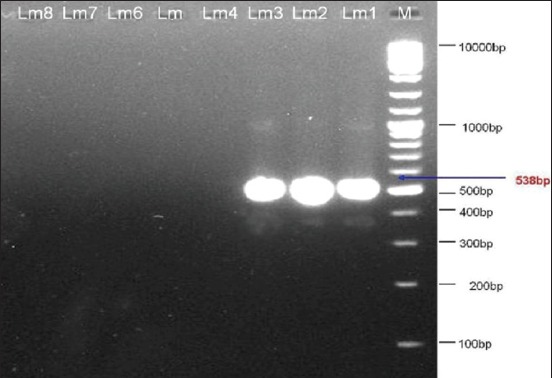
Ethidium bromide-stained agarose gel showing polymerase chain reaction products of *Listeria monocytogenes* DNA amplified with FlaAprimers. The electrophoresis performed at 80 volts for 1.25 h. Lane M, molecular-sized marker (100-10000 bp DNA ladder). Lanes Lm1-Lm3 showed amplification of *flaA* gene at 538 bp.

Three (75.0%) of the D2 positive isolates (Lm1, Lm2, and Lm3) were positive when tested with the FlaA primers, and therefore, determined to be a 1/2a, or 3a serotypes. Whereas, 1 (25.0%) isolate (Lm4) tested negative with FlaA primer set, and therefore, determined to be 1/2c or 3c (Figure-5).

Overall, serotype distribution showed that 4 isolates were serotype 4a or 4c (Lineage III), 3 isolates were serotype 1/2a or 3a (Lineage II), and only one isolate was a 1/2c or 3c serotype (Lineage II). The remaining serotypes (1/2b, 3b, 4b, 4d, and 4e) were not determined in the present study.

## Discussion

*L. monocytogenes* is known to cause listeriosis in humans and animals. No information on occurrence and distribution of *L. monocytogenes* in both veterinary and public health sectors in Najaf, Iraq, are found. Results showed that eight *L. monocytogenes* isolates were recovered from bile salts samples of sheep and cattle. We suggest that the occurrence of *Listeria* in the gallbladder of sheep and cattle was less frequent. This result was comparable with results of a survey undertaken in Al-Muthanna province, Iraq by Al-Zubaidi [12] who reported that the isolation rates of *L. monocytogenes* from gallbladder of sheep and human were 20% and 4%, respectively, but no isolate was recovered from cattle. Marien *et al*. [13] documented that only one *L. monocytogenes* isolate was recovered from the gallbladder of a dog in Belgium. No studies other than the above mentioned are available on dissemination of this species in gallbladder of animals. We suggest that the presence of a significant public health hazard is linked to the consumption of meat contaminated with *L. monocytogenes* as it is generally assumed that raw meat products cannot be free from *L. monocytogenes*. This may be due to the procedures of evisceration and food processing conducted in the local slaughterhouse of Najaf province (area of the study) that allows a greater chance of contamination. Furthermore, *Listeria* species are ubiquitous in the environment [14]. People handling food at different levels can also be sources of contamination [15,16]. According to Borch *et al*. [17], *L. monocytogenes* transmission to the carcasses does not occur primarily through the animal but is mainly linked to the slaughterhouse environment. Although there are no reports of *L. monocytogenes* infection in the area of this study, and since high fatality rate related to the infection caused by this pathogen, attention should be focused on accurate and early diagnosis of this etiological agent and disease.

In the present demonstration, the isolation rate of *L. monocytogenes* from gallbladder of sheep was more than that obtained from cattle. Al-Zubaidi [12] found that no isolate was detected in cattle compared with sheep (20%). This may be due to the fact that cows inherently less susceptible to this disease than other animals such as sheep and goats [18] and that the components of bile as cows have a number of additional bile acids comparing to human and sheep, the bile in human and sheep contain mainly cholic acid and genodeoxycholic [19]. Other salts are found in the bile components of cows at important concentrations such as saprocholic, citrolic, and litholic, and this change has importance in sensitivity of cows for infection with *L. monocytogenes* relatively less than for sheep [20]. The tradition of consuming raw or undercooked meat exacerbates the public health risk associated with *L. monocytogenes, as* Al-Zubaidi [12] reported the presence of *L. monocytogenes* in raw meat slices, ground meat, and in meat processing environments. In addition, further processing and handling of meat and coating with spices increase the risk of contamination with *Listeria* species as a final point; we support the hypothesis that gallbladders of cattle and sheep may be representing as a reservoir for human *L. monocytogenes* infections.

The frequency of *L. monocytogenes* positive gallbladder samples tends to occur during cold months. Similar results were reported by Bonardi *et al*. [21].

Specific primers were used (for *hly* gene) to confirm detection of *L. monocytogenes* isolates; the gene encoded important virulence factor (Listeriolysin O), a pore-forming exotoxin essential for invasion into the host cells and lysis of the phagosomes and that responsible for intracellular replication of *L. monocytogenes* [22]. All isolates were carried the *hly* gene, present result consistent with Jinneman and Hill [23]. The ability of *Listeria* species to produce hemolysis is closely correlated with their pathogenicity [1].

Commonly used strategies to serotypes *L. monocytogenes* strains are based on conventional and PCR methods [24,25]. However, the main objective of this study was to determine the isolates serotypes (by PCR). This study will provide baseline information and templates for practical epidemiological applications.

The amplified products for D1 (50.0%) and D2 (50.0%) were detected in the isolates. Borucki and Call [5] reported that D1 and D2 primers differentiated serotypes 1/2b, 3b, and serotype 4 strains from serotypes 1/2a, 1/2c, 3a, and 3c. The four isolates identified as belonging in Division I/III (D1) were subtyped using primers designed to differentiate serotypes 4 and 1/2b/3b. These primers called Glt were designed from a 1/2b serotype specific region flanking the *gltA-B* cassette described by Lei *et al*. [26]. However, the four isolates did not give the expected PCR product size with Glt primers. The isolates negative to Glt were subjected to PCR using Mama primers. Isolates tested positive with these primers were identified as 4a or 4c.

All the four isolates gave positive results with Mama specific primers. The present study revealed that serotype 4b was not detected since the four D1 positive isolates were also positive with Mama specific primers. However, serotype 4b has been found to be the main serotype associated with human listeriosis [27].

The four isolates identified as belonging to Division II (D2) were serotyped using primers designed from the *flaA* gene, which encodes the *L. monocytogenes* flagellin protein [28]. Isolates that tested positive with the FlaA primers were considered as serotype 1/2a or 3a, while isolates tested negative were considered as serotype 1/2c or 3c [5]. Through the study, serotypes distribution showed that out of 4 isolates, three were belonged to serotypes 1/2a or 3a, while 1 isolate belonged to serotype 1/2c or 3c.

Most studies found serotypes 1/2a and 1/2b as the most common serotypes in food [29,30], a finding that is also supported in studies by Gilot *et al*. [31]. Wallace *et al*. [30] found serotype 1/2a in 90% of all the *L. monocytogenes* isolates tested in food samples. One ambition of present study was to identify the presence of serotype 1/2a or 3a which is the number one among the *L. monocytogenes* serotypes associated with human listeriosis [27]. However, this serotype was positively detected. This serotype has also been detected in foodstuffs in the previous study [27]. It was observed that *L. monocytogenes* serotypes 1/2a, 1/2b, and 4b are responsible for 98% of documented human listeriosis cases [32]. The presence of 1/2a serotype in bile salt samples indicated a risk factor to infect the human through cross-contamination of meat in slaughterhouse, and this was consistent with Sjoman [33] who observed that serotype 1/2a was the most common for human infections during 1990-2001 in Finland.

## Conclusion

We concluded that gallbladders of cattle and sheep may be representing a pool for human *L. monocytogenes* infection and the presence of 1/2a serotype indicates that there is a risk for human infection through cross-contamination of meat in slaughterhouses in the location of the study.

## Authors’ Contributions

We declare that this work was done by the authors named in this article and all liabilities pertaining to claims relating to the content of this article will be borne by the authors. HJA and MAA are main supervisors of the study, organized the data, and responsible for interpretation of the results. SAA and ZJH collected the samples from the slaughterhouses. AHA and AMA were responsible for the laboratory data analysis. All authors read and approved the final manuscript.
